# A high-resolution history of the South American Monsoon from Last Glacial Maximum to the Holocene

**DOI:** 10.1038/srep44267

**Published:** 2017-03-10

**Authors:** Valdir F. Novello, Francisco W. Cruz, Mathias Vuille, Nicolás M. Stríkis, R. Lawrence Edwards, Hai Cheng, Suellyn Emerick, Marcos S. de Paula, Xianglei Li, Eline de S. Barreto, Ivo Karmann, Roberto V. Santos

**Affiliations:** 1 Instituto de Geociências, Universidade de São Paulo, São Paulo 05508-090, Brazil; 2 Department of Atmospheric and Environmental Sciences, University at Albany, Albany, New York 12222, USA; 3 Departamento de Geoquímica,Universidade Federal Fluminense, Niterói, Rio de Janeiro 24220-900, Brazil; 4 Department of Earth Sciences, University of Minnesota, Minneapolis, Minnesota 55455, USA; 5 Institute of Global Environmental Change, Xi’an Jiaotong University, Xi’an 710049, China; 6 Instituto de Geociências, Universidade de Brasília, Brasília, Brazil

## Abstract

The exact extent, by which the hydrologic cycle in the Neotropics was affected by external forcing during the last deglaciation, remains poorly understood. Here we present a new paleo-rainfall reconstruction based on high-resolution speleothem δ^18^O records from the core region of the South American Monsoon System (SAMS), documenting the changing hydrological conditions over tropical South America (SA), in particular during abrupt millennial-scale events. This new record provides the best-resolved and most accurately constrained geochronology of any proxy from South America for this time period, spanning from the Last Glacial Maximum (LGM) to the mid-Holocene.

Changes in the tropical hydrologic cycle during the transition from the LGM to the Holocene have so far not been explored in greater detail over tropical SA. Similarly, our understanding of millennial-scale variability and abrupt changes in South American precipitation during this transition from glacial to interglacial conditions is still rudimentary. The longest paleoclimate records available^1^,^2^,^3^,^4^,^5^ do not present a chronology and resolution that would allow exploring individual millennial-scale events, while the few high-resolution records in existence only cover limited time intervals^6^,^7^.

Current interpretations focus primarily on the role of high-latitude forcing, mainly Northern Hemisphere (NH) temperature, as being responsible for abrupt changes in precipitation over tropical SA^5^,^6^. This link between tropical SA precipitation and high-latitude temperature is established through changes in the latitudinal position of the Inter-tropical Convergence Zone (ITCZ), which responds to changes in the inter-hemispheric energy balance. Thus, the ITCZ is shifted southward in response to NH cooling and the resulting anomalous cross-equatorial temperature gradient^8^. This meridional temperature gradient is driven mainly by changes in NH temperature during millennial-scale events, since changes in northern high-latitude temperature during these events reached approximately 10 °C over the course of decades^9^, whereas changes in Antarctic temperature occurred more gradually over the course of several centuries with a smaller amplitude of only 1° to 3 °C^10^.

The influence of Antarctic temperature on tropical SA precipitation on the other hand is not nearly as clear^6^. Southern Hemisphere (SH) millennial-scale events documented in the isotopic record from Antarctica, such as the Antarctic Isotope Maxima events (AIMs), or the Antarctic Cold Reversal (ACR) may potentially have affected precipitation in tropical SA, as these events have been documented in glacial records from the Andes as well as in some other proxies from southern SA^6^,^10^,^11^. These southern teleconnections, however, have not yet been properly tested and documented using paleo-precipitation records from the tropical lowlands.

Austral summer precipitation over tropical and sub-tropical SA is related to the SAMS^12^,^13^, a distinct feature of SA climate, which, unlike the narrowly confined ITCZ, penetrates far south over the continent during the summer months (Fig. 1). Oceanic and coastal precipitation related to the ITCZ reaches its southernmost location and peak precipitation during austral autumn (MAM), while the mature phase of the SAMS with maximum precipitation occurs during the summer months DJF, in relation with the formation of the South Atlantic Convergence Zone (SACZ). The SACZ^14^ is defined as an elongated convective band, originating over the southeastern Amazon basin, and extending over southeastern Brazil into the southwest Atlantic (Fig. 1). Previous studies have documented that both SACZ and ITCZ positions have shifted in the past in response to abrupt millennial-scale events, thereby significantly affecting SAMS precipitation over the SA continent^7^ and leading to an anti-phased relationship with NH monsoon systems^5^.

Here, we present a new paleo-rainfall record based on oxygen isotopes in speleothems. This record contains the most highly-resolved data and the most accurately constrained geochronology of any proxy from SA for the time period spanning from the Last Glacial Maximum (LGM) to the Holocene. It is located in central South America (Brazil), in the core of the SAMS domain (Fig. 1), at a location where hitherto no proxy records of similar resolution have been recovered. Fornace *et al*.^15^ recently published a stable isotopic record derived from leaf waxes in lake sediments from a shallow lake located at the Brazil-Bolivia border, a few hundred kilometers to the northwest from our site. Their record covers the past 40,000 years, but at a very low resolution (~1000 years), insufficient to resolve millennial-scale variability.

## Results

### Cave location and amount effect

**Figure 1:**
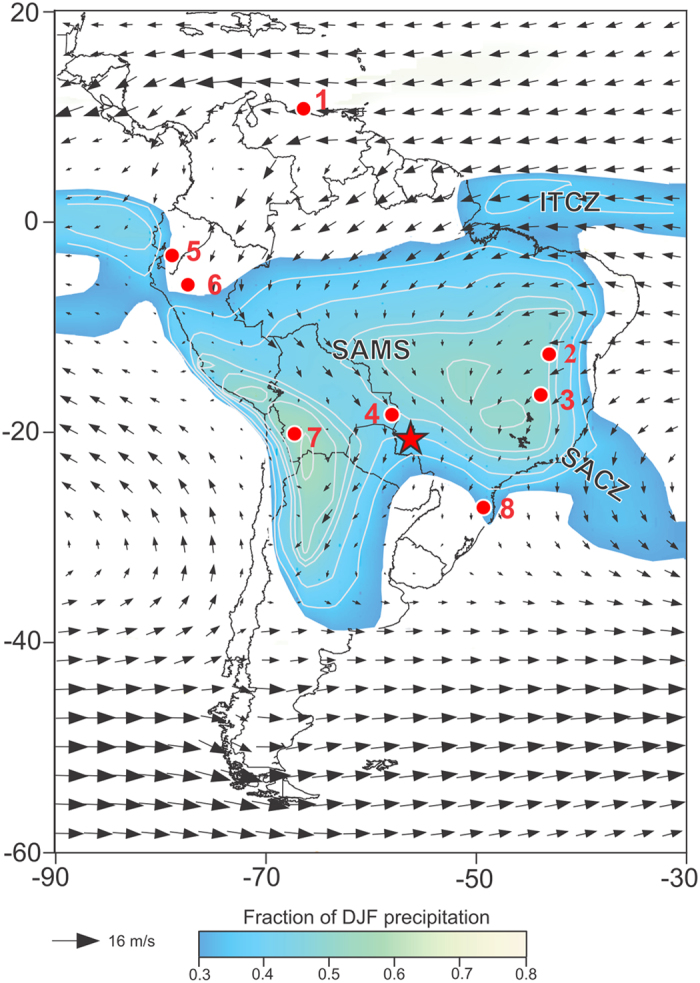
Map of South America with the locations of the records discussed in text. Austral summer (DJF) 850 hPa wind field and fractional DJF precipitation. Color shading indicates regions where fraction of total annual precipitation falling during austral summer (DJF) >0.3, highlighting the extent of the SAMS over the continent; contour interval is 0.05. Wind data is from ERA-Interim and precipitation data from GPCC, with averages calculated over period 1979–2014. Red star indicates the location of Jaraguá cave (our JAR record); 1- Cariaco Basin^27^; 2- Paixão cave^7^; 3- Lapa Sem Fim cave^7^; 4- Laguna La Gaiba^15^,^23^; 5- Santiago Cave^4^; 6- El Condor and Cueva del Diamante caves^5^; 7- Salar Uyuni^1^; 8- Botuverá Cave^2^. The figure was created using the software Adobe Illustrator CS6 version 16.0.0, in similar way as in Novello *et al*.^18^.

The stalagmites presented in this study were collected in the Jaraguá cave (21°05′S, 56°35′W, ~570 m above sea level), located in Bonito City, State of Mato Grosso do Sul, Brazil (Fig. 1). The climate in Bonito City is tropical with a three month long dry season during austral winter (JJA). Between September 2011 and December 2014 we monitored rainfall amount, isotopes in rainfall and air temperature in the region of Jaraguá cave. Based on these data, we establish that the isotopic composition of rainfall (δ^18^O) at our site is anti-correlated with precipitation amount on seasonal timescales. In the atmosphere, the δ^18^O of the condensate decreases with decreasing air temperature^16^, but the seasonal cycles of temperature and δ^18^O composition of precipitation in the tropics are often opposed to each other. That is, high (low) temperatures coincide with more depleted (enriched) δ^18^O values^17^, as shown by our results (Supplementary Fig. S1). This phenomenon is interpreted in the sense that the δ^18^O composition of precipitation reflects precipitation amount rather than temperature, because at low latitudes high temperatures and rainy seasons tend to coincide^17^. Novello *et al*.^18^ recently documented, based on 30 years of measurements performed at a nearby station (Cuiabá) by the International Atomic Energy Agency (IAEA) that this anti-correlation between δ^18^O and precipitation amount also holds on interannual timescales, confirming earlier results from the same location by Vuille *et al*.^17^. They further documented that this relationship extends significantly upstream, suggesting that δ^18^O at this site is also suited to track the degree of rainout and hence monsoon intensity over a larger domain, consistent with similar results for the past two millennia^19^. The δ^18^O and δD measured in rainfall near our cave site also shows a good correspondence with the Global Meteoric Water Line (Supplementary Fig. S2) indicating that evaporation processes do not significantly influence the isotopic fractionation at our study site. In summary, the results from our monitoring support the use of δ^18^O as a proxy for local- and regional-scale monsoon-season rainfall amount. This interpretation is in agreement with model simulations over tropical and subtropical areas of South America^17^ and with the current paleoclimatic interpretation of speleothem records at locations within the SAMS domain^3^,^4^,^5^,^6^,^7^,^18^,^19^,^20^,^21^.

The mean air temperature, averaged over the monitored period inside of the cave is 21.4 °C with an annual amplitude of 1.4 °C, in stark contrast to the annual temperature amplitude of 47.8 °C outside the cave (Supplementary Fig. S3). The temperature range measured inside the cave during the monitoring period may at most cause a fractionation in δ^18^O in speleothem calcite of ~0.3‰^22^, very close to the instrumental analytical error. The cave temperature is representative of the mean annual temperature of the external environment and since the isotopic fractionation factor in calcite is dependent on temperature^22^, the temperature change over longer time scales needs to be considered when interpreting δ^18^O. A temperature reconstruction at a nearby site^23^ estimated that temperature during the late Pleistocene and Holocene was close to modern values (~26 °C). For the last glacial period, the record indicates that temperatures were 4 °C colder with a relatively large uncertainty. A cooling of 4 °C at Jaraguá cave, would affect the δ^18^O in our JAR record by less than 1‰^21^ during the LGM, when compared to the Holocene.

### Speleothem δ^18^O record

**Figure 2:**
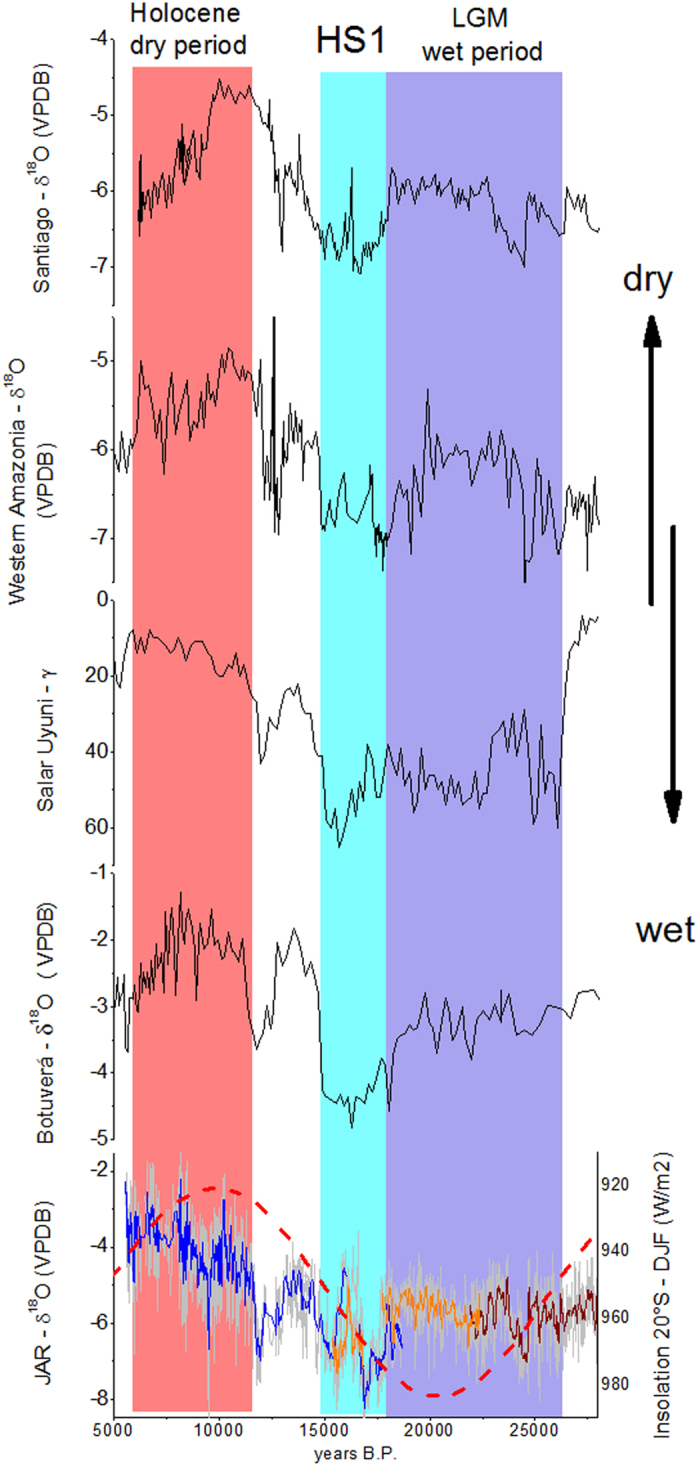
Speleothem δ^18^O record from Jaraguá cave in comparison with other South American climate records. Comparison of δ^18^O data from stalagmites JAR7 (blue), JAR14 (orange) and JAR13 (wine) with other paleo-rainfall records from South America: Botuverá cave^2^; Salar Uyuni^1^; caves from western Amazonia^5^ and Santiago cave^4^, also in western Amazonia.

The Jaraguá cave record (JAR – Fig. 2) is composed of 3390 δ^18^O measurements from 3 stalagmites (JAR7, JAR14, JAR13, Supplementary Figs S4–S6) and 80 U-Th ages (Supplementary Table S1) linearly interpolated, covering a time interval between 5,550 BP (considering 2014 CE to be year 0 BP) and 27,970 BP. We discarded three ages in JAR7 because they presented relatively high errors and were out of stratigraphic order (see Supplementary Table S1). The resulting record has an average resolution varying between 6.8 and 10.0 years, making this data set arguably the highest-resolution and geochronologically best-constrained paleoclimate archive from SA for the period covering the transition from the LGM to the Holocene. The number of isotopic measurements performed on each speleothem, as well as the number of U-Th ages obtained, are listed in Supplementary Table S2 together with the average resolution and period covered by each sample. Note that stalagmite JAR14 overlaps with stalagmite JAR7 from 15,395 BP to 16,870 BP and from 17,600 BP to 18,660 BP and with stalagmite JAR13 from 21,915 BP to 22,370 BP.

## Discussion

**Figure 3:**
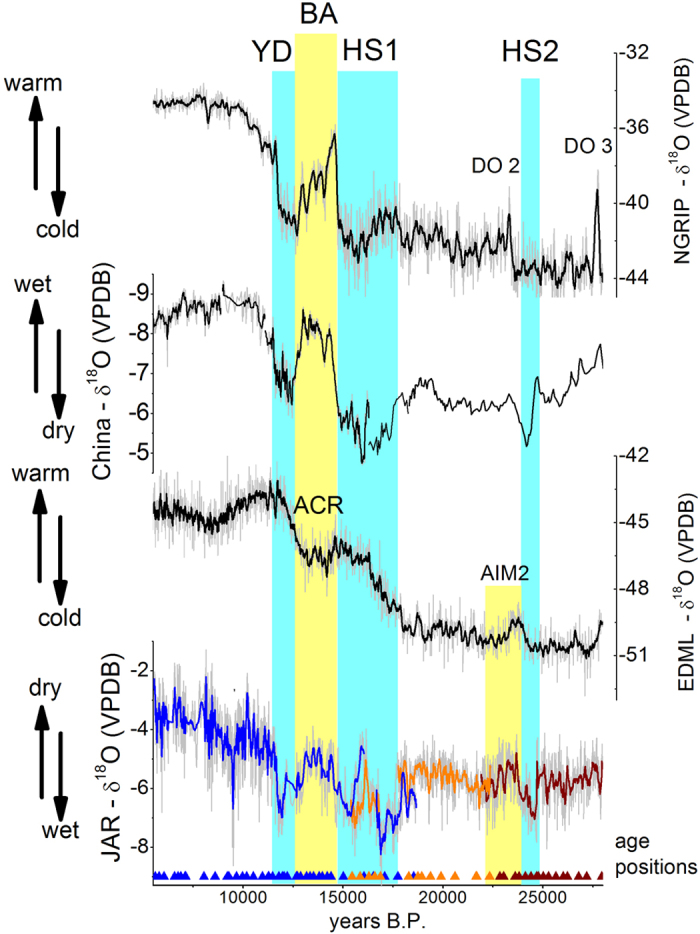
Comparison of the speleothem δ^18^O record from Jaraguá cave with other climate records. Comparison between JAR record (11-point running mean) and EDML ice core δ^18^O (7-point running mean) from Antarctica^31^, Chinese speleothem δ^18^O records (inverted scale – 4 point running mean) from Hulu and Dongge caves^32^,^33^,^34^,^35^ and NGRIP δ^18^O ice core record (7-point running mean) from Greenland^36^. δ^18^O data from JAR record composed by stalagmites JAR7 (blue), JAR14 (orange) and JAR13 (wine).

The JAR record has a mean δ^18^O value of −5.2‰ with a range between −1.3‰ and −9.3‰. Our record indicates wetter conditions during the last glacial period (17,800–27,970 BP; mean δ^18^O value is −5.7‰) when compared to the early and mid-Holocene (5,550–11,000 BP; mean δ^18^O value is −3.9‰). This tendency for a drier Holocene is consistent with other paleo-rainfall records from the western^1^,^4^,^5^ and southeastern^2^,^3^ portions of the SAMS domain (Fig. 2). It is also consistent with the relatively high (low) austral summer insolation in the SH during the LGM (Holocene) leading to enhanced (subdued) convective activity in the southern tropics, as borne out in model simulations for the LGM and the Holocene^24^. The comparison between our JAR record and stalagmite records of the NH monsoon in China further document this hemispherically anti-phased behaviour of tropical precipitation and monsoon systems in response to insolation forcing on precessional time scales (Fig. 3).

The fact that the JAR record is characterized by wetter conditions during the Last Glacial than during the Holocene is in disagreement with interpretations of biological proxies at a nearby site^15^,^23^. However, vegetation in the region may not have responded primarily to changes in precipitation. Given that our record of increased precipitation during the LGM is consistent with similar palaeo-rainfall records further northwest over the western Amazon^4^,^5^ and Andes^6^ and to the southeast over southern Brazil^3^, indicates that moisture was enhanced across Brazil, from northwest to southeast, throughout the last glacial period.

**Figure 4:**
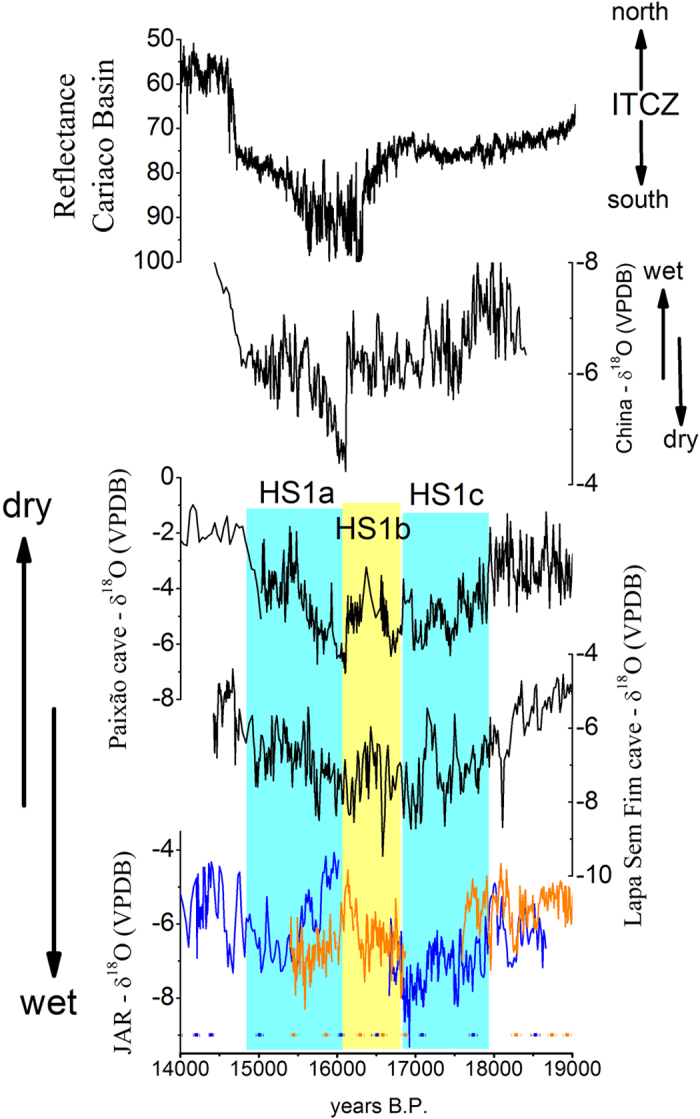
Comparison of paleoclimate records documenting the HS1 event. Comparison between JAR δ^18^O record and Lapa Sem Fim and Paixão δ^18^O records^7^, Chinese δ^18^O records from Qingtian Cave^26^ and reflectance of sedimentary core record from Cariaco Basin^27^.

A number of abrupt millennial-scale events characterize the transition from LGM to the Holocene in the JAR record. Anomalies during the two youngest Heinrich stadial events (HS1 and HS2) as well as the Bølling-Allerød (BA) and the Younger Dryas (YD) periods can clearly be identified (Fig. 3). In our record, the period between 23,800–24,700 BP that corresponds to HS2, is characterized by δ^18^O values that are depleted by approximately 1.6‰, compared to prior and posterior conditions (Supplementary Fig. S7). The period of 12,900–14,700 BP, which corresponds to the BA period, on the other hand is characterized by a δ^18^O increase of 2.4‰, followed by a decrease of 2.2‰ heading into the period that corresponds to the YD (11,600–12,900 BP, Fig. 3). The wet conditions during HS2 and the YD, as well as the drier conditions during the BA recorded in our JAR record are clearly due to NH high latitude forcing. The cold temperatures in the NH during HS2 and the YD lead to a southward displacement of the ITCZ, thereby increasing the moisture influx into SA, while at the same time weakening the NH monsoons (Fig. 3). Opposite conditions likely prevailed during the BA (Fig. 3). During the period that corresponds to HS1 (~17,730–14,800 BP), however, the JAR14 sample shows a particular feature, with a double-plunge structure containing two wet periods, as indicated by the δ^18^O decrease of ~3.2‰ during the intervals ~17,730–16,840 BP and 16,040–14,800 BP. These two wet phases are separated by a dry excursion, which does not have an equivalent counterpart in NH records (Fig. 4). This dry phase shown in JAR14 is concomitant with the lack of deposition (hiatus) in the stalagmite JAR7, which occurs between 16,051 ± 39 BP and 16,513 ± 80 BP. Isotopic values between 15,395–16,045 BP are offset by ~200 years in the JAR7 record, when compared to JAR14. This is likely due to an absence of chronological tie-points at this time period or due to the relatively low resolution between 15,012 and 16,051 BP in the JAR7 sample.

The two wet phases of HS1 in SA, separated by this slightly drier period, were previously reported, albeit with a smaller amplitude (Fig. 4), in the δ^18^O records from Paixão and Lapa Sem Fim caves^7^ located in the easternmost part of the SAMS domain (Fig. 1). In that study, the dry phase half-way through HS1 was attributed to a temporary northward displacement of the ITCZ, due to a warmer tropical North Atlantic. Consistent with this hypothesis, a paleoclimate record in Central America, sensitive to moisture and temperature, indicates a small wet perturbation during this intermediate phase of HS1^25^. However, this episode is only marginally evident in NH monsoon records or other proxies recording the position of the ITCZ. For example, the combined Asian monsoon δ^18^O record from Hulu and Dongge caves shows a minor wet event during the interval corresponding to HS1, but with much reduced amplitude when compared to the more humid conditions during the LGM and BA (Fig. 3). Zhang *et al*.^26^ presented another East Asian monsoon δ^18^O record, with better resolution and geochronology for this event, consistent with the position of the ITCZ as recorded by the sedimentary record from the Cariaco Basin^27^ (Fig. 4). After ~19,000 BP the NH monsoon slowly weakens in the same way that the SAMS strengthens in the SH, but around ~16,850 BP the SAMS intensity starts to decrease, independently of a change in the ITCZ position, thereby losing its relationship with the NH monsoonal circulation (Fig. 4). Thus, the dry phase during HS1 recorded in tropical Brazilian records appears to be a characteristic of the SAMS that is only weakly dependent of high latitude climate conditions and ITCZ position.

The beginning of the cold event HS2 in the NH is accompanied by an abrupt shift in our δ^18^O record to more negative values at 24,700 BP (Fig. 3). The JAR record also shows a short dry event in the middle of the HS2 period, centred at 24,250 BP, which is concomitant with a northward position of ITCZ and warming of Greenland (Supplementary Fig. S7). Subsequently, the δ^18^O increases progressively until 23,650 BP, reaching values similar to the period prior to HS2, before dropping again until 22,200 BP. Over this period, the SAMS record is qualitatively similar to the Antarctic EDML ice core temperature (δ^18^O) record during Antarctic Isotope Maximum (AIM) event 2 (Supplementary Fig. S7), however, it is difficult to verify this similarity quantitatively over this short interval. Our record is also consistent with the positioning of the ITCZ recorded over the Cariaco Basin (Supplementary Fig. S7). Interestingly, Kanner *et al*.^6^ show a similar close correspondence between the SAMS and Antarctic temperature during AIM8 and AIM12, based on a speleothem record from Peru, consistent with our record during AIM2. Kanner *et al*.^6^ also linked the behavior of the SAMS during these events to latitudinal variations in the position of the ITCZ, but the exact mechanism that links the ITCZ with Antarctic temperature is still not fully understood. If Antarctic temperature were to significantly affect the inter-hemispheric temperature gradient, the response of the SAMS during AIM2 should be the exact opposite of what is recorded at Jaraguá cave. The abrupt NH forcing, as documented in the IRD record during HS2 (between 24,250–22,200 BP) and the subsequent warm period during DO2 recorded in δ^18^O from Greenland, on the other hand, are consistent with the wet and dry periods of the SAMS, respectively (Supplementary Fig. 7), suggesting that even during the AIM events the SAMS felt the influence of changes in NH temperature.

During the Antarctic Cold Reversal (ACR), our record again seems to more closely follow NH temperature, with drier conditions in response to NH warming during the BA (Fig. 3). Jomelli *et al*.^11^ reported glacial advances in the tropical Andes during that time. Our record suggest that the SAMS was weakened and that therefore glaciers advanced likely due to colder temperatures associated with the ACR, rather than due to increased snowfall associated with enhanced available moisture from the SAMS. This interpretation is consistent with the colder conditions during the ACR reported from almost one hundred mid- and high-latitudes sites in the SH^10^.

Our new record shows that the LGM period was wetter in SA when compared to the early and mid-Holocene, which is in agreement with climatic conditions reconstructed from palaeo-rainfall records in northwestern and southeastern SA. When combined, these isotopic records document that rainfall conditions changed in a coherent way across the entire monsoon belt from the Andes to southeastern Brazil during the transition from the LGM to the Holocene. Vegetation reconstructions from southern and central Amazonia^15^,^23^,^28^, which show an expansion of vegetation from the LGM to the Holocene, may thus the vegetation have responded primarily to changes in temperature or amount of CO_2_ in the atmosphere, both parameters that increased during the transitions LGM to Holocene. The JAR record also shows that millennial-scale abrupt events were important features of the SAMS, characterizing the hydrological cycle over tropical SA during the transition from the LGM to the Holocene. NH temperature forcing, which is generally considered to act as the main control knob for SAMS variability on a number of different time scales, is evident in the JAR δ^18^O record during DO2, HS2, the BA and the YD. During HS1, however, our record exhibits an accentuated dry phase, separating two wet periods at the beginning and end of HS1, which does not have a strong corresponding counterpart in any high latitude or NH proxies.

## Methods

The amount of precipitation and air temperature outside of the cave were recorded by a Hobo Pendant Event Data Logger (model UA-003–64), while temperature and relative humidity inside the cave were recorded by a Hobo Temperature/Relative Humidity Data Logger (model U23–001). Rainwater for isotopic analysis was sampled every 15 days in a water collector installed together with the rain gauge.

The analyses for δ^18^O and δD of water were performed using a Picarro L2120-i Analyzer at the Stable Isotope Laboratory of the University of Brasilia. The data are reported as Vienna Standard Mean Ocean Water (VSMOW) standard with precision of ±0.1‰ (δ^18^O) and ±0.5 (δD). For carbonate speleothems, the oxygen isotope ratios are expressed in δ notation, the per mil deviation from the Vienna Peedee Belemite (VPDB) standard. For each measurement, approximately 100 μg of powder was drilled from the sample and analyzed with an on-line, automated, carbonate preparation system linked to a Thermo-Finnigan Delta Plus Advantage at the Centro de Pesquisas Geocronológicas of the Geosciences Institute of Universidade de São Paulo (CPGeo-IGc-USP). The speleothem reproducibility of standard materials is 0.1‰ for δ^18^O.

The stalagmites were dated by U-Th method at the Isotope Laboratory of the University of Minnesota (USA) and Xi’an Jiaotong University (China). The powder carbonate samples (~100 mg) on stalagmites were drilled using a carbide dental drill following stratigraphic horizons. To separate uranium and thorium the chemical procedure described in Edwards *et al*.^29^ was applied. After the separation of uranium and thorium each sample was dried and diluted for injection into the spectrometer. The analysis was performed using a multi-collector inductively coupled plasma mass spectrometry technique in a MC-ICP-MS, Thermo-Finnigan NEPTUNE, according to the techniques described in Cheng *et al*.^30^.

## Additional Information

**How to cite this article:** Novello, V. F. *et al*. A high-resolution history of the South American Monsoon from Last Glacial Maximum to the Holocene. *Sci. Rep.* **7**, 44267; doi: 10.1038/srep44267 (2017).

**Publisher's note:** Springer Nature remains neutral with regard to jurisdictional claims in published maps and institutional affiliations.

## Supplementary Material

Supplementary Material
