# Supplementary material for: A high-resolution history of the South American Monsoon from Last Glacial Maximum to the Holocene
Source: Sci Rep. 2017 Mar 10;7:44267. doi: 10.1038/srep44267 (PMC5345026; doi:10.1038/srep44267)
Supplement: Supplementary Material [file srep44267-s1.pdf]

Supplementary Information for

A high-resolution history of the South American Monsoon from Last Glacial Maximum to the Holocene

Valdir F. Novello[1]*, Francisco W. Cruz[1], Mathias Vuille[2], Nicolás M. Stríkis[3], R. Lawrence Edwards[4], Hai Cheng[4,5], Suellyn Emerick[1], Marcos S. de Paula[1], Xianglei Li[5], Eline de S. Barreto[1], Ivo Karmann[1], Roberto V. Santos[6]

[1]Instituto de Geociências, Universidade de São Paulo, São Paulo 05508-090, Brazil. [2]Department of Atmospheric and Environmental Sciences, University at Albany, Albany, New York 12222, USA. [3]Departamento de Geoquímica,Universidade Federal Fluminense, Niterói, Rio de Janeiro 24220-900, Brazil.[4]Department of Earth Sciences, University of Minnesota, Minneapolis, Minnesota 55455, USA. [5]Institute of Global Environmental Change, Xi'an Jiaotong University, Xi'an 710049, China. [6]Instituto de Geociências, Universidade de Brasília, Brasília, Brazil.

(*) Corresponding author: Valdir Felipe Novello, Instituto de Geociências, Universidade de São Paulo, Rua do Lago, 562, CEP 05508-080, São Paulo, Brazil. (vfnovello@gmail.com).

This document includes:

Figures S1 to S7

Table S1 and S2

References

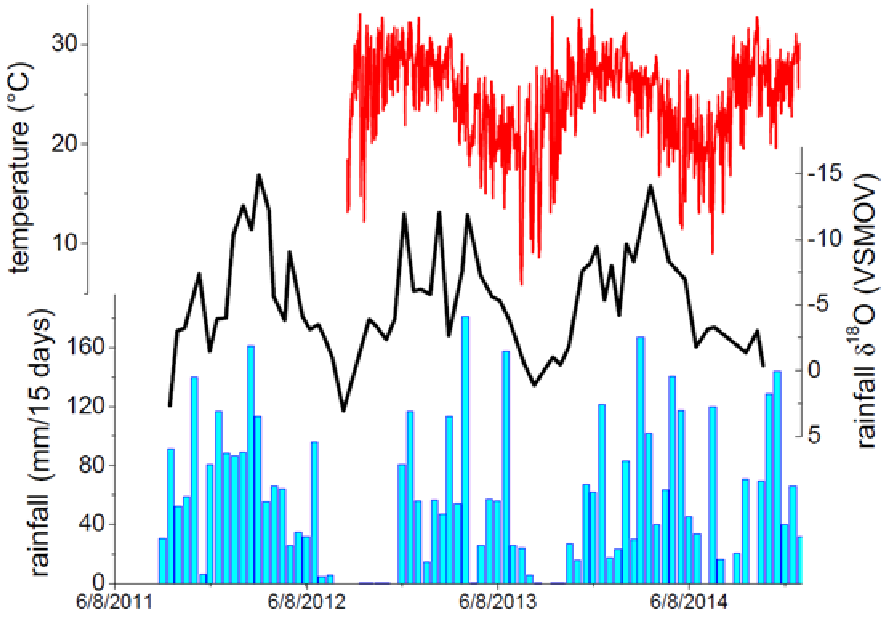

Figure S1. Comparison between biweekly (15 day) accumulated precipitation (blue), rainfall $\delta^{18}O$ (black) and daily mean air temperature (red). Sampling site located close to Jaraguá cave. Note that scale for rainfall $\delta^{18}O$ is inverted.

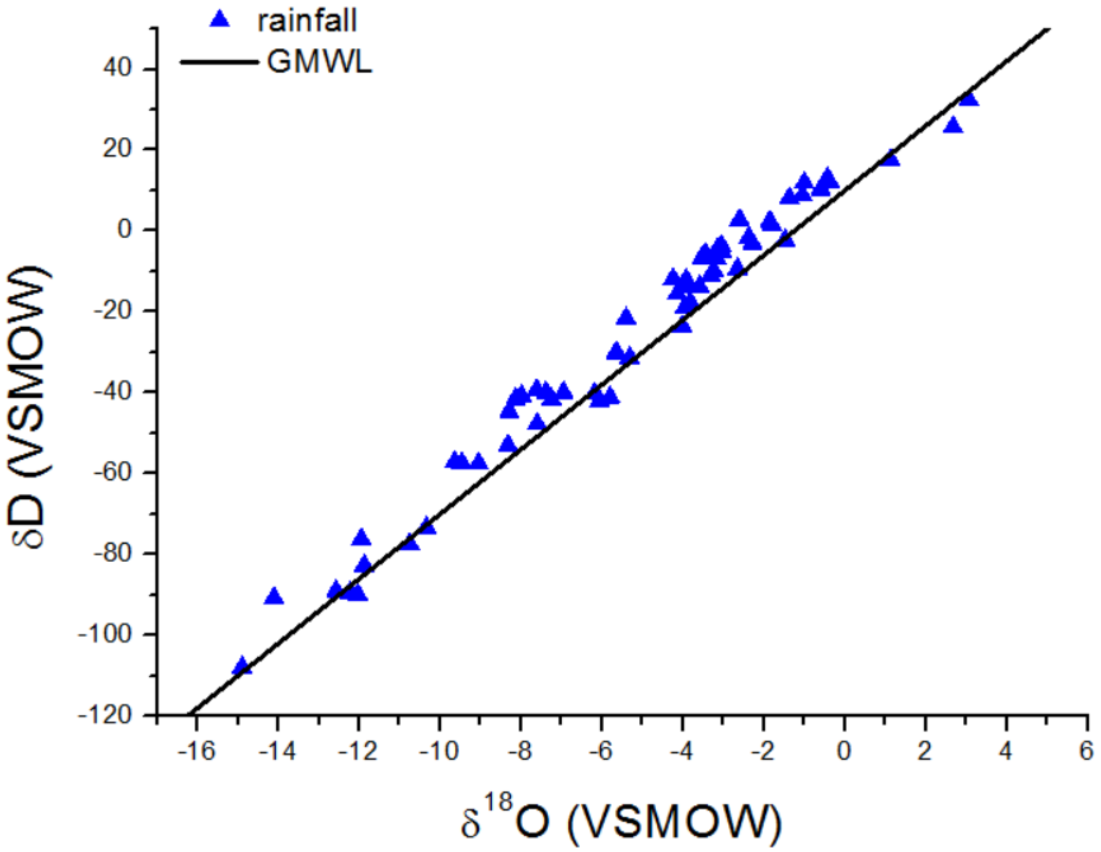

Figure S2. Relationship between δD and $\delta^{18}O$ of rainfall at sampling site located close to Jaraguá cave, plotted alongside the Global Meteoric Water Line[1].

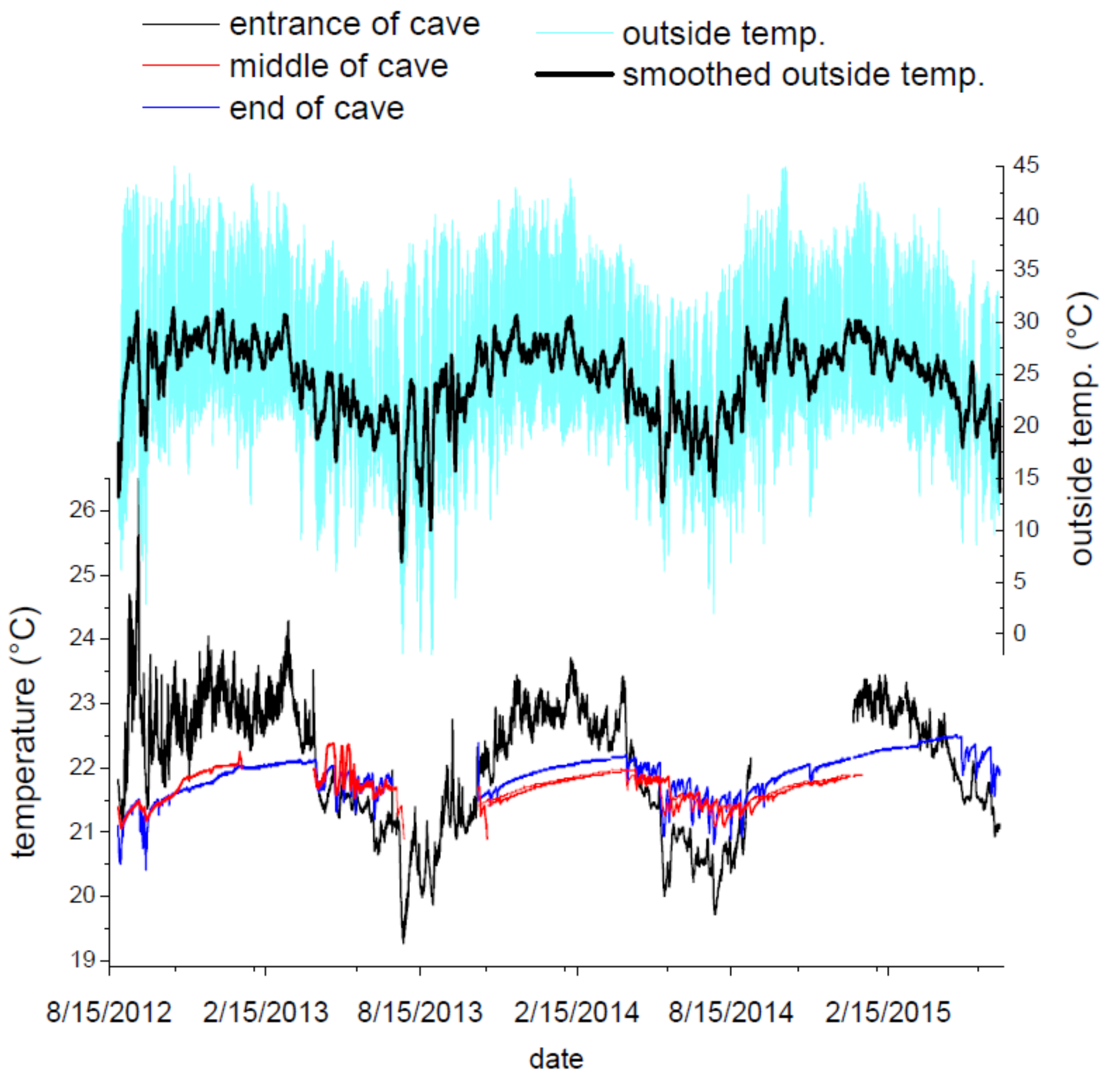

Figure S3. Bottom: Air temperature monitored at the entrance (black), middle (red) and end (blue) of Jaraguá cave. Top: Outside air temperature hourly (light blue) and the smoothed average (black).

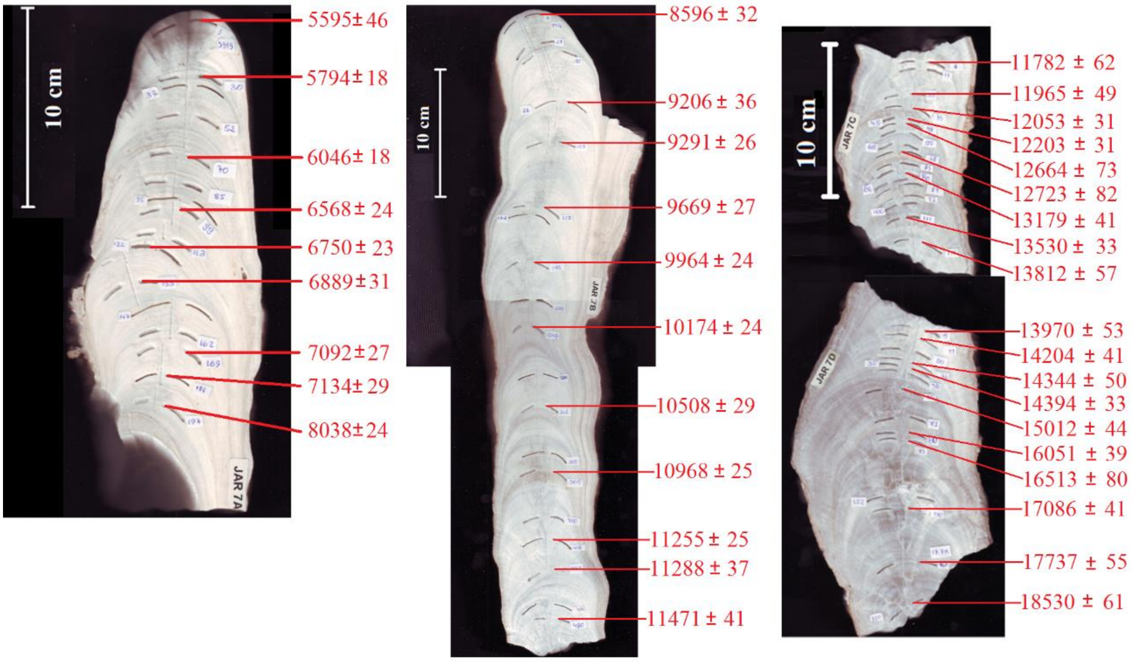

Figure S4. Stalagmite JAR7 with the position of U/Th ages.

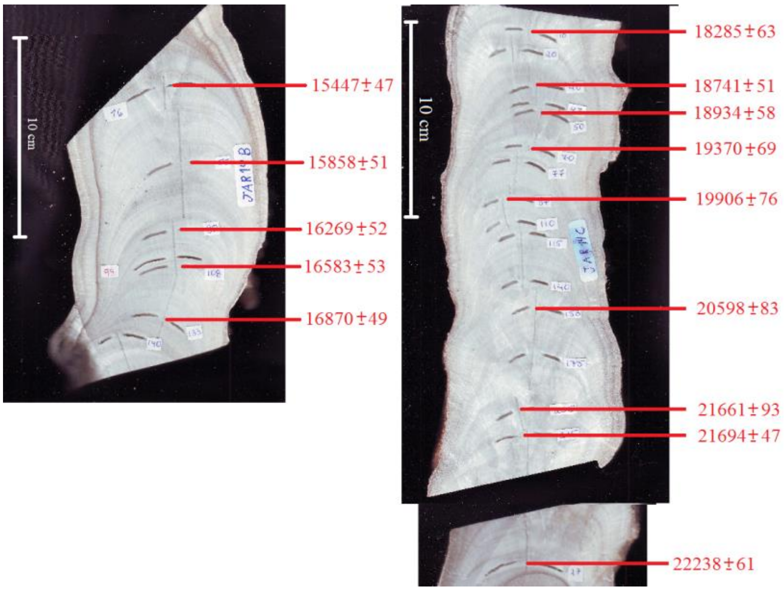

Figure S5. Stalagmite JAR14 with the position of U/Th ages.

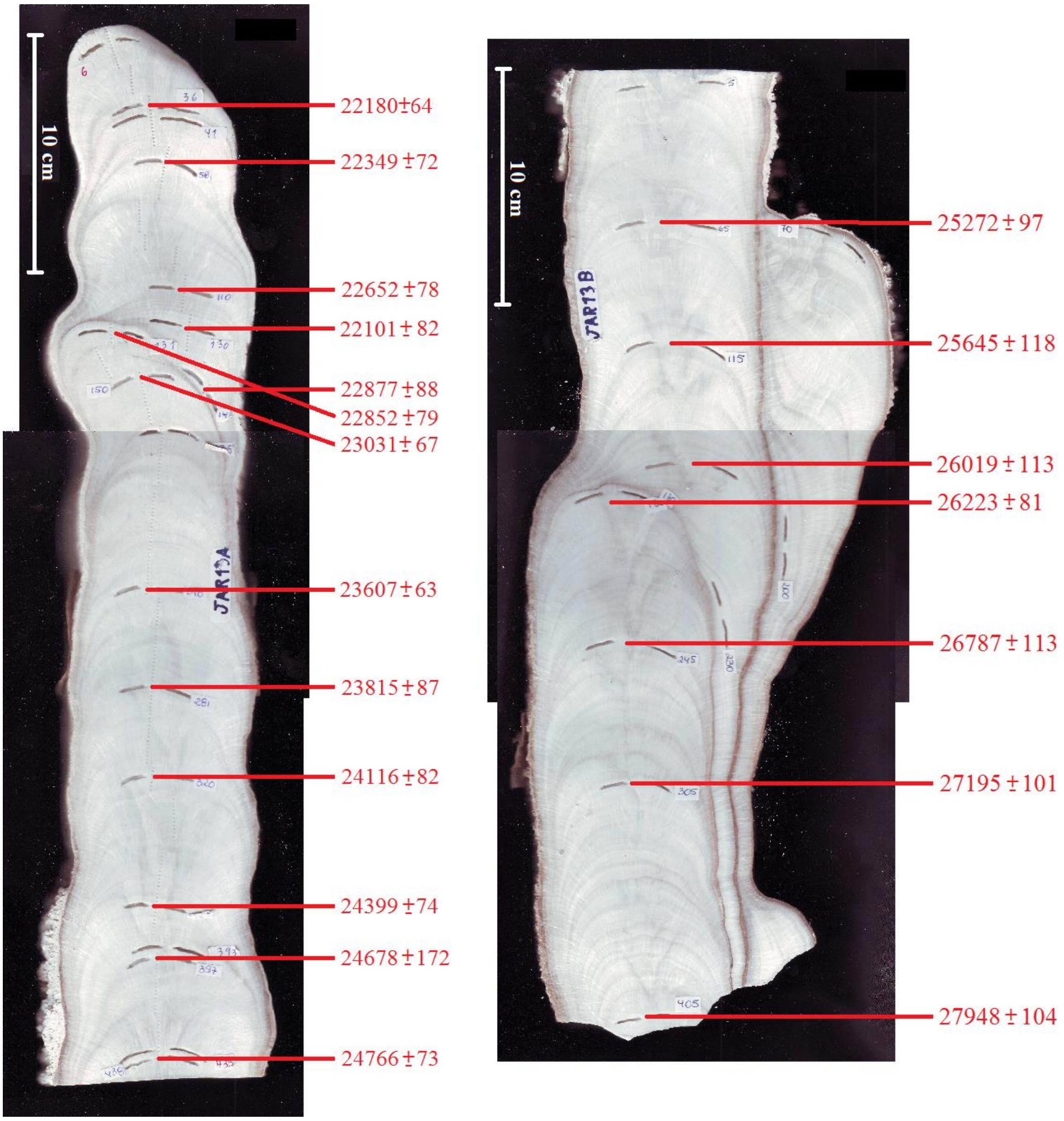

Figure S6. Stalagmite JAR13 with the position of U/Th ages.

Table S1

Sample name	positions in relationship to top (mm)	^{238}U (ppb)	^{232}Th (ppt)	^{230}Th / ^{232}Th (atomic x10^{-6})	d^{234}U* (measured)	^{230}Th / ^{238}U (activity)	^{230}Th Age (yr) (uncorrected)	^{230}Th Age (yr) (corrected)	d^{234}U$_{Initial}$** (corrected)	
**JAR7A-5**	4.9	1013 ± 5	184 ± 5	5300 ± 134	167 ± 5	0.05854 ± 0.00038	5,600 ± 46	**5,595 ± 46**	170 ± 6	
**JAR7A-30**	37.7	3779 ±9	307 ±6	12500 ±256	186.9 ±1.7	0.0615 ±0.0002	5796 ±18	**5794 ±18**	190 ±2	
**JAR7A-70**	73.4	6526 ±15	107 ±3	64087 ±1493	185.4 ±1.5	0.0640 ±0.0002	6046 ±18	**6046 ±18**	189 ±2	
**JAR7A-99**	101.2	7161 ±22	399 ±8	21163 ±434	220.9 ±1.7	0.0715 ±0.0002	6570 ±24	**6568 ±24**	225 ±2	
**JAR7A-122**	121.6	4062 ±10	468 ±9	10369 ±210	204.7 ±1.7	0.0725 ±0.0002	6753 ±23	**6750 ±23**	209 ±2	
**JAR7A-135**	139.8	6899 ±25	238 ±5	34517 ±721	176.9 ±2.0	0.0722 ±0.0003	6889 ±31	**6889 ±31**	180 ±2	
**JAR7A-169**	175.6	6810 ±22	191 ±4	44296 ±929	196.9 ±1.8	0.0755 ±0.0003	7092 ±27	**7092 ±27**	201 ±2	
**JAR7A-185**	188.9	2426 ±7.0	109 ±3	27945 ±817	205.3 ±2.0	0.0765 ±0.0003	7135 ±29	**7133 ±29**	209 ±2	
**JAR7A-197**	203.8	4102 ±9	212 ±4	26741 ±551	178.1 ±1.5	0.0839 ±0.0002	8039 ±24	**8038 ±24**	182 ±2	
**JAR7B-4**	324.3	2195 ±4,7	732 ±15	4600 ±93	224,2 ±2,2	0,0931 ±0,0003	8604 ±31	**8596 ±32**	230 ±2	
**JAR7B-74**	391.3	2183 ±4	3184 ±425	1142 ±152	239.8 ±1.6	0.1010 ±0.0002	9240 ±27	**9206 ±36**	246 ±2	
**JAR7B-103**	426.6	3217 ±6	394 ±8	13188 ±269	195.5 ±1.6	0.0979 ±0.0002	9294 ±26	**9291 ±26**	201 ±2	
**JAR7B-155**	478	2532 ±5	373 ±8	11765 ±239	235.2 ±1.6	0.1051 ±0.0003	9672 ±27	**9669 ±27**	242 ±2	
**JAR7B-199**	522.6	3233 ±5	577 ±12	10020 ±204	237.9 ±1.5	0.1084 ±0.0002	9968 ±24	**9964 ±24**	245 ±2	
**JAR7B-248**	572.9	1924 ±4	574 ±12	6089 ±123	233.2 ±1.7	0.1102 ±0.0003	10181 ±28	**10174 ±29**	240 ±2	
**JAR7B-312**	637	1947 ±4	527 ±11	7056 ±143	257.3 ±1.6	0.1159 ±0.0003	10514 ±29	**10508 ±29**	265 ±2	
**JAR7B-365**	684.7	1907 ±3	231 ±5	16353 ±338	250.1 ±1.5	0.1200 ±0.0002	10971 ±25	**10968 ±25**	258 ±2	
**JAR7B-420**	740	1270 ±2	140 ±3	19128 ±395	296.6 ±1.5	0.1276 ±0.0002	11258 ±25	**11255 ±25**	306 ±2	
**JAR7B-422**	760.6	1392 ±3	609 ±12	4807 ±99	289.9 ±1.7	0.1274 ±0.0003	11298 ±36	**11288 ±37**	299 ±2	
**JAR7B-470**	798.4	1057 ±2,1	485 ±10	4774 ±97	322,9 ±2,1	0,1327 ±0,0004	11481 ±41	**11471 ±41**	334 ±2	
**JAR7C-6**	842.4	3148 ±3	9678 ±194	710 ±14	280.4 ±1.3	0.1323 ±0.0004	11851 ±38	**11782 ±62**	290 ±1	
**JAR7C-33**	864	2303 ±5	431 ±9	11945 ±254	300.0 ±2.1	0.1357 ±0.0005	11969 ±49	**11965 ±49**	310 ±2	
**JAR7C-35**	873.6	3032 ±3	768 ±16	8828 ±190	291.0 ±1.5	0.1357 ±0.0003	12059 ±31	**12053 ±31**	301 ±2	
**JAR7C-45**	880.5	1533 ±3	375 ±8	9336 ±189	301.8 ±1.6	0.1384 ±0.0003	12209 ±31	**12203 ±31**	312 ±2	
**JAR7C-48**	883.8	148.7 ±0.1	52 ±2	6729 ±202	306.2 ±1.6	0.1439 ±0.0008	12672 ±73	**12664 ±73**	317 ±2	discarted age
**JAR7C-65**	897.6	1139 ±2	130 ±3	20486 ±446	304.2 ±1.5	0.1416 ±0.0003	12483 ±31	**12481 ±31**	315 ±2	
**JAR7C-68**	903	103.8 ±0.1	57 ±1	4328 ±105	302.0 ±2.0	0.1441 ±0.0008	12735 ±82	**12723 ±82**	313 ±2	
**JAR7C-80**	916.6	1310 ±2	272 ±6	11194 ±236	261.4 ±1.6	0.1411 ±0.0004	12883 ±40	**12879 ±40**	271 ±2	
**JAR7C-89**	924.6	88.8 ±0.1	68 ±2	3214 ±83	265.3 ±1.6	0.1492 ±0.0011	13626 ±106	**13608 ±107**	276 ±2	discarted age
**JAR7C-100**	927.6	1413 ±3	256 ±5	13002 ±277	250.0 ±1.9	0.1429 ±0.0004	13183 ±41	**13179 ±41**	259 ±2	
**JAR7C-111**	946.9	977 ±1	234 ±5	10129 ±208	257.0 ±1.5	0.1473 ±0.0003	13535 ±33	**13530 ±33**	267 ±2	
**JAR7C-127**	1106.9	524 ±0.7	726 ±15	1792 ±37	258.9 ±1.7	0.1507 ±0.0005	13842 ±53	**13810 ±57**	269 ±2	
**JAR7D-5**	989	2079 ±4	521 ±11	10017 ±210	261.7 ±1.8	0.1523 ±0.0005	13970 ±53	**13964 ±53**	272 ±2	
**JAR7D-11**	992.4	2041 ±2	1336 ±27	3896 ±80	260.0 ±1.3	0.1547 ±0.0004	14219 ±40	**14204 ±41**	271 ±1	
**JAR7D-32**	1009.9	2620 ±2	1483 ±31	4477 ±94	240.7 ±1.2	0.1537 ±0.0005	14357 ±49	**14344 ±50**	251 ±1	
**JAR7D-35**	1013.4	1218 ±2	472 ±10	6592 ±133	247.2 ±1.4	0.1549 ±0.0003	14403 ±33	**14394 ±33**	257 ±1	
**JAR7D-50**	1027.8	2139 ±4	567 ±11	9925 ±201	235.8 ±1.6	0.1596 ±0.0004	15018 ±44	**15012 ±44**	246 ±2	
**JAR7D-80**	1056.6	1829 ±3	336 ±7	15528 ±316	257.8 ±1.4	0.1730 ±0.0003	16055 ±39	**16051 ±39**	270 ±1	
**JAR7D-85**	1061.9	172.8 ±0.2	40 ±1	12642 ±410	246.8 ±1.4	0.1761 ±0.0008	16518 ±80	**16513 ±80**	259 ±2	
**JAR7D-122**	1097.4	2956 ±7	7894 ±9857	1041 ±1300	269.9 ±1.9	0.1686 ±0.0062	15458 ±611	**15397 ±617**	282 ±2	discarted age
**JAR7D-130**	1106.9	3705 ±3	1542 ±32	7346 ±152	271.2 ±1.3	0.1854 ±0.0004	17095 ±40	**17086 ±41**	285 ±1	
**JAR7D-167**	1142	2494 ±4.5	555 ±11	14085 ±285	14085 ±285	259.1 ±1.8	17742 ±55	**17737 ±55**	272 ±2	
**JAR7D-200**	1173.4	2681 ±5	499 ±12	17405 ±409	17405 ±409	0.1964 ±0.0005	18534 ±61	**18530 ±61**	263 ±2	

Stalagmite JAR14 (14 analysis)									
**Sample name**	**positions in relationship to the first sampled point top (mm)**	**^{238}U (ppb)**	**^{232}Th (ppt)**	**^{230}Th / ^{232}Th (atomic x10^{-6})**	**$d^{234}U$* (measured)**	**^{230}Th / ^{238}U (activity)**	**^{230}Th Age (yr) (uncorrected)**	**^{230}Th Age (yr) (corrected)**	**$d^{234}U_{Initial}$** (corrected)**
**JAR14B-16**	5.5	3970.3 ±5.6	371 ±9	28077 ±712	199.8 ±1.8	0.1591 ±0.0003	15449 ±41	**15447 ±41**	209 ±2
**JAR14B-55**	45	3223 ±8	371 ±7	23680 ±480	215.3 ±1.6	0.1652 ±0.0004	15861 ±51	**15858 ±51**	225 ±2
**JAR14B-90**	78	2301 ±5	3055 ±61	2159 ±43	244.3 ±1.5	0.1738 ±0.0004	16327 ±47	**16296 ±52**	256 ±2
**JAR14B-108**	107.8	1978 ±4	483 ±10	11675 ±245	220.9 ±1.7	0.1730 ±0.0005	16589 ±53	**16583 ±53**	231 ±2
**JAR14B-133**	131.2	1657 ±3	133 ±3	35937 ±819	214.0 ±1.5	0.1747 ±0.0004	16872 ±49	**16870 ±49**	224 ±2
**JAR14C-10**	141.7	2228 ±5	994 ±20	7139 ±145	244.3 ±1.9	0.1932 ±0.0005	18296 ±63	**18285 ±63**	257 ±2
**JAR14C-40**	170.7	1480 ±2	525 ±11	9828 ±200	329.1 ±1.6	0.2113 ±0.0005	18749 ±51	**18741 ±51**	347 ±2
**JAR14C-50**	182.7	1648 ±3	169 ±4	33098 ±724	287.4 ±1.8	0.2064 ±0.0005	18936 ±58	**18934 ±58**	303 ±2
**JAR14C-70**	202.7	1825 ±4	406 ±8	15180 ±311	250.6 ±2.2	0.2047 ±0.0006	19375 ±69	**19370 ±69**	265 ±2
**JAR14C-97**	229.7	1484 ±3	213 ±5	23809 ±528	236.2 ±1.8	0.2074 ±0.0007	19909 ±76	**19906 ±76**	250 ±2
**JAR14C-153**	286.7	1778 ±4	285 ±6	21865 ±457	229.6 ±2.5	0.2128 ±0.0006	20602 ±83	**20598 ±83**	243 ±3
**JAR14C-205**	336.7	2877 ±6	1280 ±26	8281 ±171	232.5 ±1.7	0.2234 ±0.0008	21672 ±92	**21661 ±93**	247 ±2
**JAR14C-215**	347.2	2208 ±3	528 ±11	15553 ±314	242.0 ±1.3	0.2254 ±0.0004	21700 ±47	**21694 ±47**	257 ±1
**JAR14D-27**	373.7	3177.7 ±4.6	837 ±18	14131 ±302	212.8 ±1.7	0.2258 ±0.0005	22333 ±60	**22327 ±61**	227 ±2

Stalagmite JAR13 (23 analyisis)									
**Sample name**	**positions in relationship to the first sampled point top (mm)**	**^{238}U (ppb)**	**^{232}Th (ppt)**	**^{230}Th / ^{232}Th (atomic x10^{-6})**	**$d^{234}U$* (measured)**	**^{230}Th / ^{238}U (activity)**	**^{230}Th Age (yr) (uncorrected)**	**^{230}Th Age (yr) (corrected)**	**$d^{234}U_{Initial}$** (corrected)**
**JAR13A-36**	37.5	4438.3 ±7.5	155 ±7	94394 ±4053	81.7 ±1.6	0.1996 ±0.0004	22180 ±64	**22180 ±64**	87 ±2
**JAR13A-58**	61.5	3215 ±7	1136 ±23	9530 ±192	99.2 ±1.5	0.2043 ±0.0005	22359 ±72	**22349 ±72**	106 ±2
**JAR13A-110**	120	4582 ±12	211 ±4	73507 ±1532	90.7 ±1.5	0.2051 ±0.0006	22653 ±78	**22652 ±78**	97 ±2
**JAR13A-130**	136.5	5739 ±12	648 ±14	29171.8 ±652.3	86.5 ±1.8	0.1998 ±0.0006	22105 ±82	**22101 ±82**	92 ±2
**JAR13A-145**	154.5	3461 ±9	606 ±13	19756 ±410	105.3 ±1.7	0.2099 ±0.0007	22882 ±88	**22877 ±88**	112 ±2
**JAR13A-131**	160.04	2635 ±3.4	317 ±9	28513 ±794	95.5 ±1.5	0.2077 ±0.0006	22855 ±79	**22852 ±79**	102 ±2
**JAR13A-150**	184	3486 ±7	407 ±8	29430 ±604	90.9 ±1.3	0.2083 ±0.0005	23034 ±67	**23031 ±67**	97 ±1
**JAR13A-240**	276.84	2553 ±5	287 ±6	31212 ±643	89.0 ±1.3	0.2126 ±0.0004	23610 ±63	**23607 ±63**	95 ±1
**JAR13A-281**	318.4	3699 ±9	189 ±4	69157 ±1469	89.8 ±1.9	0.2144 ±0.0006	23816 ±87	**23815 ±87**	96 ±2
**JAR13A-320**	356.8	3602 ±9	188 ±4	68655 ±1430	95.1 ±1.5	0.2179 ±0.0006	24117 ±82	**24116 ±82**	102 ±2
**JAR13A-375**	411.2	4316 ±8	596 ±12	26279 ±534	94.4 ±1.6	0.2201 ±0.0005	24402 ±74	**24399 ±74**	101 ±2
**JAR13A-397**	435.2	3456 ±10	267 ±15	46748 ±2653	78.5 ±1.8	0.2190 ±0.0013	24680 ±172	**24678 ±172**	84 ±2
**JAR13A-438**	322.4	3320 ±7	262 ±5	46299 ±956	87.7 ±1.4	0.2216 ±0.0005	24768 ±73	**24766 ±73**	94 ±1
**JAR13B-5**	495.4	4245 ±13	623 ±13	25088 ±512	85.2 ±1.8	0.2231 ±0.0007	25024 ±104	**25020 ±104**	91 ±2
**JAR13B-65**	554.6	4090 ±12	264 ±6	57768 ±1215	90.3 ±1.8	0.2262 ±0.0007	25274 ±105	**25272 ±105**	97 ±2
**JAR13B-115**	605.8	3896 ±13	290 ±6	50979 ±1061	96.4 ±2.1	0.2305 ±0.0008	25647 ±118	**25645 ±118**	104 ±2
**JAR13B-170**	658.6	3912 ±12	265 ±6	56852 ±1190	94.9 ±1.9	0.2332 ±0.0008	26021 ±113	**26019 ±113**	102 ±2
**JAR13B-174**	209.6	3897.1 ±7.5	125 ±6	117741 ±6033	87.3 ±1.9	0.2299 ±0.0006	25822 ±88	**25821 ±88**	94 ±2
**JAR13B-183**	710.9	4170.9 ±7.2	222 ±7	71865 ±2379	80.2 ±1.7	0.2315 ±0.0005	26224 ±81	**26223 ±81**	86 ±2
**JAR13B-245**	772.4	3983 ±10	235 ±5	66364 ±1345	85.2 ±1.6	0.2371 ±0.0007	26788 ±100	**26787 ±100**	92 ±2
**JAR13B-305**	832.4	4153 ±10	252 ±5	64928 ±1315	80.1 ±1.7	0.2391 ±0.0007	27197 ±101	**27195 ±101**	87 ±2
**JAR13B-338**	863.9	3391.9 ±6.1	487 ±11	27388.2 ±645.6	81.1 ±1.9	0.238465 ±0.00056	27088 ±92	**27084 ±92**	88 ±2
**JAR13B-405**	932.9	3762 ±10	619 ±12	24449 ±494	76.1 ±1.8	0.2440 ±0.0007	27953 ±104	**27948 ±104**	82 ±2

$^{\alpha}\delta^{234}U = ([^{234}U/^{238}U]$activity - 1) x 1000.

$^{\beta}\delta^{234}U$initial corrected was calculated based on ^{230}Th age (T), i.e., $\delta^{234}U$initial = $\delta^{234}U$measured X $e^{\lambda_{234} * T}$, and T is corrected age.

[c] $[^{230}Th/^{238}U]_{activity} = 1 - e^{-\lambda 230T} + (\delta^{234}U_{measured}/1000)[\lambda 230/(\lambda_{230} - \lambda_{234})](1 - e^{-(\lambda_{230} - \lambda_{234}) T})$, where T is the age.

Decay constants are 9.1577×10^{-6} yr^{-1} for ^{230}Th, 2.8263×10^{-6} yr^{-1} for ^{234}U, and 1.55125×10^{-10} yr^{-1} for ^{238}U (Cheng et al., 2000).

[d] The degree of detrital ^{230}Th contamination is indicated by the $[^{230}Th/^{232}Th]$ atomic ratio instead of the activity ratio.

[e] Age corrections were calculated using an average crustal $^{230}Th/^{232}Th$ atomic ratio of $4.4 \times 10^{-6} \pm 2.2 \times 10^{-6}$.

Those are the values for a material at secular equilibrium, with the crustal $^{232}Th/^{238}U$ value of 3.8. The errors are arbitrarily assumed to be 50%.

sample name	Number of isotopic analyses	Number of U-Th ages	mean resolution (years)	Period covered (years BP*)
**JAR7**	1924	43	6.8	5550 - 18660
**JAR14**	863	14	8	15395 - 22370
**JAR13**	603	23	10	21915 - 27970

Table S2. Number of isotopic analyses and U-Th ages, resolution and period covered by the stalagmites that compose the JAR record. (*) The year of 2014 (C.E) is considered the present year.

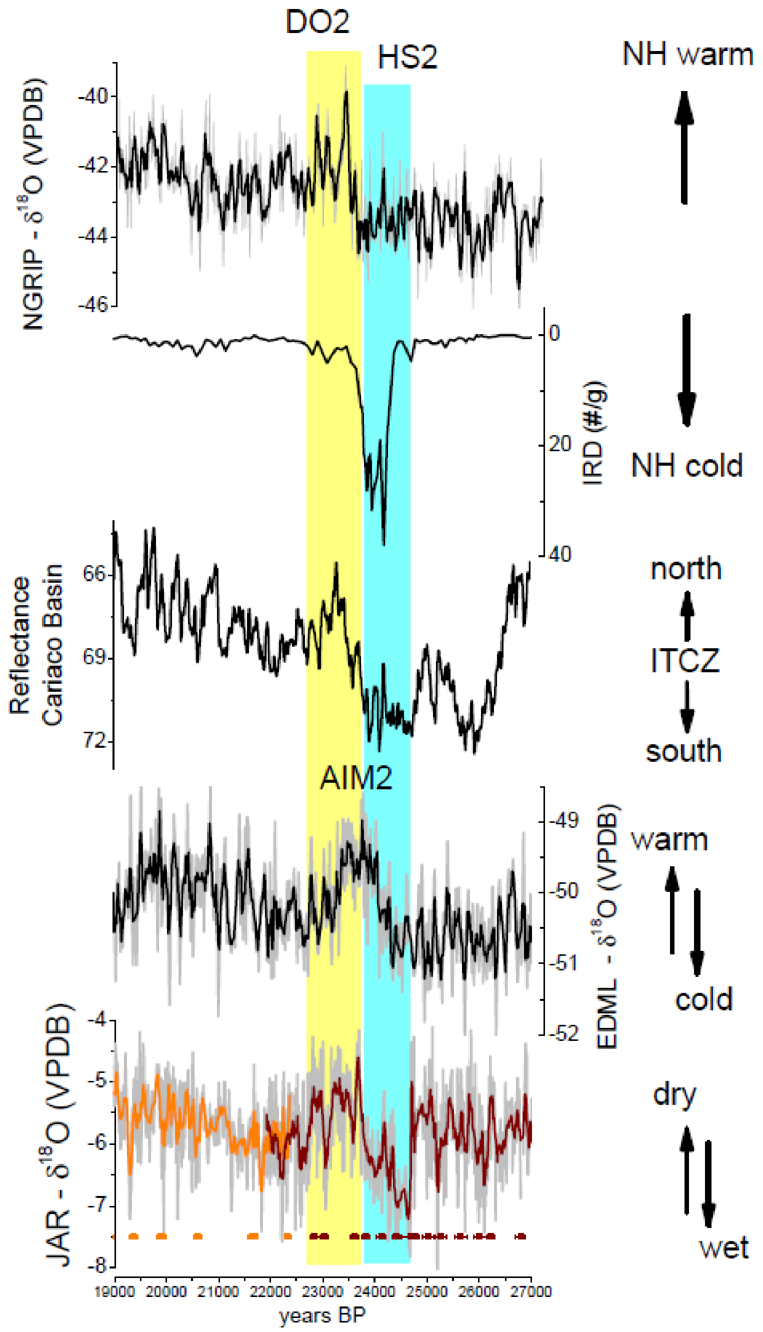

Figure S7. Comparison of paleoclimate records documenting the AIM2 and HS2 events. Detailed comparison of AIM2 recorded in JAR record (smooth line represents 9-point running mean) with EDML ice core (3-point running men) from Antarctica[2], reflectance of sedimentary core record from Cariaco Basin[3], Ice-Rafted Debris (IRD) content in ocean sediment[4] and NGRIP $\delta^{18}O$ ice core record (3-point running mean) from Greenland[5].

References

1. Dansgaard, W. Stable isotopes in precipitation. *Tellus* **16**, 436-468 (1964).
2. EPICA Community Members: Stable oxygen isotopes of ice core EDML, PANGAEA, doi:10.1594/PANGAEA.754444 (2010).
3. Deplazes, G. et al. Links between tropical rainfall and North Atlantic climate during the last glacial period. *Nat. Geosci.* **6**, 213-217 (2013).
4. Abreu, L., Shackleton, N. J., Schönfeld, J., Hall, M. & Chapman, M. Millennial-scale oceanic climate variability off the Western Iberian margin during the last two glacial periods. *Mar. Geol.* **196**, 1-20, doi:10.16/S0025-3227(03)00046-X (2003).
5. NGRIP members. High-resolution climate record of the northern hemisphere reaching into the last interglacial period. *Nature* **431**, 147–151 (2004).
